# Robotic microlaryngeal surgery: a new retractor that provides improved access to the glottis

**DOI:** 10.1186/s40064-016-1788-2

**Published:** 2016-02-27

**Authors:** Jennifer P. Rodney, Nilesh R. Vasan

**Affiliations:** Department of Otorhinolaryngology, The University of Oklahoma Health Sciences Center, PO Box 26901, Oklahoma, OK 73126-0901 USA

## Abstract

Robotic surgery has become the standard of care for many procedures outside of otolaryngology, and now is gaining momentum within our specialty. The robot has several advantages to human hands, including removal of tremor and better access to lesions due to increased degree of movement of the articulated instruments. The glottis has rarely been addressed using robotics because access was previously thought to be difficult. We present a case report using the modular oral retractor system to perform robotic microlaryngeal surgery.

## Background

**Fig. 1:**
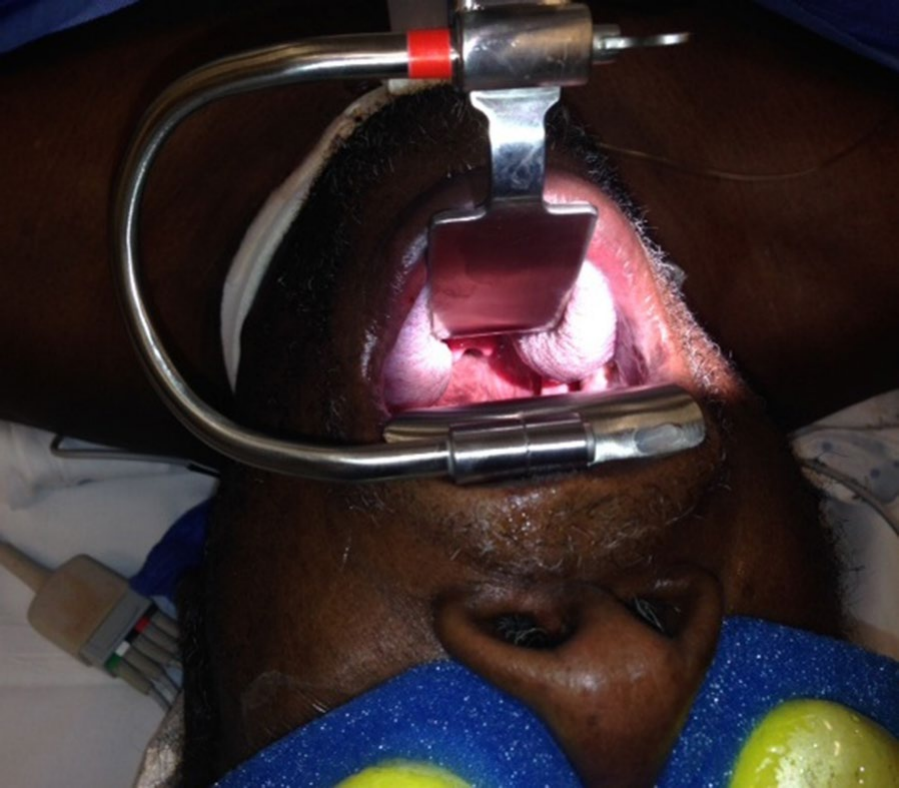
The MOR system positioned for robotic microlaryngeal surgery

Robotic surgery has become more common for many surgical procedures. It eliminates human tremor and can be less invasive than open procedures, often resulting in decreased hospital stay and faster recovery (Dogan et al. 2001; Menon et al. 2003). Unfortunately, the larynx has remained a difficult anatomical area to address with robotic surgery, limited by the size and space requirements of current robotic instrumentation. Transoral robotic surgery (TORS) has steadily been gaining ground in otolaryngology, specifically for oropharyngeal and supraglottic resections for both benign and malignant neoplasms as well as for sleep-disordered breathing. The larynx has remained a difficult problem area in robotic surgery, however, limited by the size and space requirements of the robotic arms. A small number of studies have assessed the potential of RMLS, all using different methods of exposure. A recurring theme during these investigations is that multiple instruments have to be used to obtain access to the glottis and space for the robotic instruments is limited (Dogan et al. 2001; Hockstein et al. 2005a, b). Traditional glottic surgery is limited to direct laryngoscopes, which do not allow wide field access to glottic lesions. Visualization is limited to the small view provided through the laryngoscope using a microscope. We have developed a device, called the modular oral retractor (MOR) system that is able to easily obtain a view of the glottis with the robot and maximize space in the glottic region to allow the robotic arms to function (Fig. 1). The device will enable RMLS to be less cumbersome and a more reasonable option when compared to traditional methods of glottic surgery.

## Case report

A 56 year-old man with a long history of smoking initially presented with progressive hoarseness and dysphagia for 8 months.  He underwent direct laryngoscopy and biopsy at an outside facility which showed squamous hyperplasia of the bilateral true vocal folds and diffuse, severe supraglottic hypertrophy. Nasopharyngoscopy performed in our clinic revealed redundant supraglottic mucosa that prolapsed into the glottic airway with associated plicae ventricularis, retroflexed epiglottis and prolapsed aryepiglottic folds. It was not possible to view the glottis due to the redundant mucosa. The remainder of the head and neck exam was unremarkable. The patient was deemed a good candidate for robotic-assisted surgery using the MOR system. Informed consent was obtained for examination of the larynx and removal of the obstructing mucosa with a CO_2_ laser. He was consented for use of the MOR system under an IRB-approved protocol.

## Procedure

**Fig. 2:**
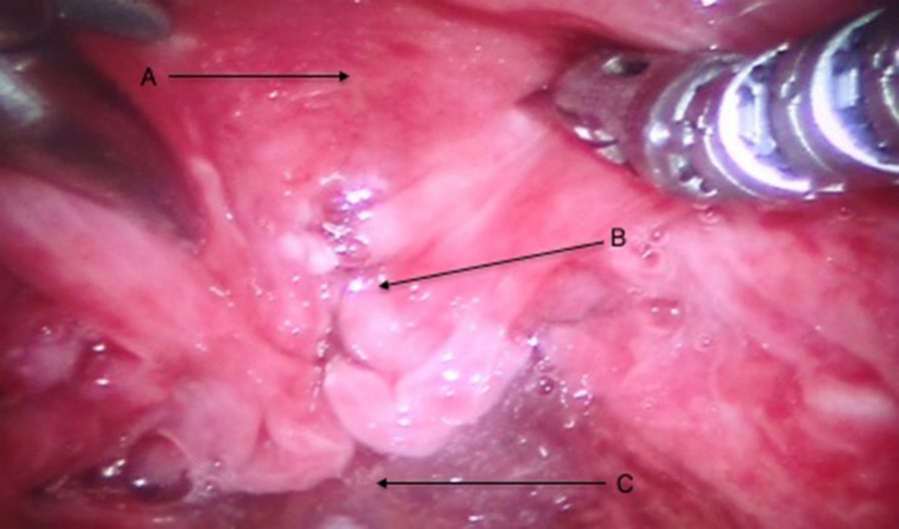
Intraoperative photo; *A* laryngeal surface of the epiglottis; *B* redundant false ventricular tissue overlying the vocal folds; *C* postcricoid area

**Fig. 3:**
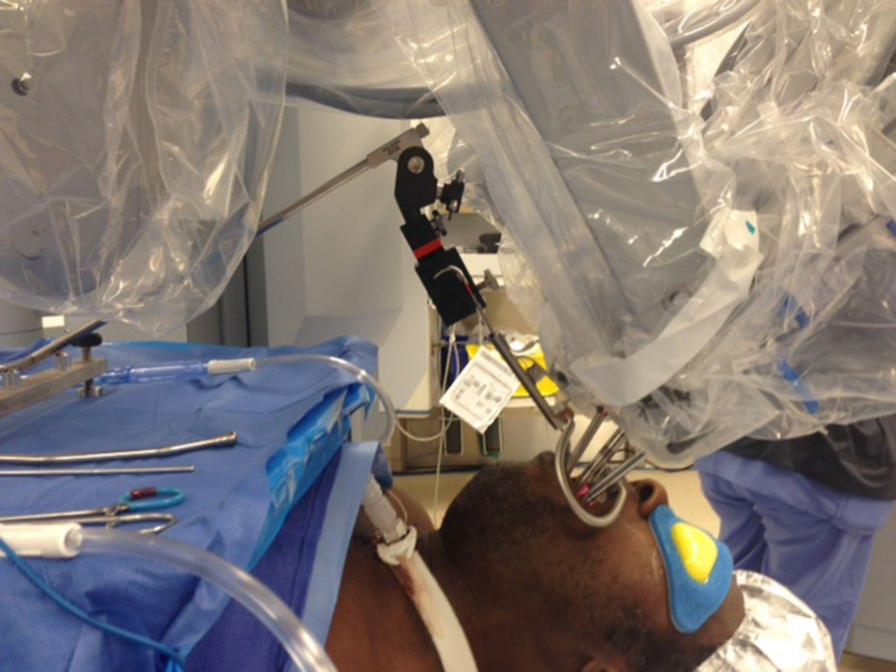
The robot and the MOR system in position. 225 × 169 mm (72 × 72 DPI)

**Fig. 4:**
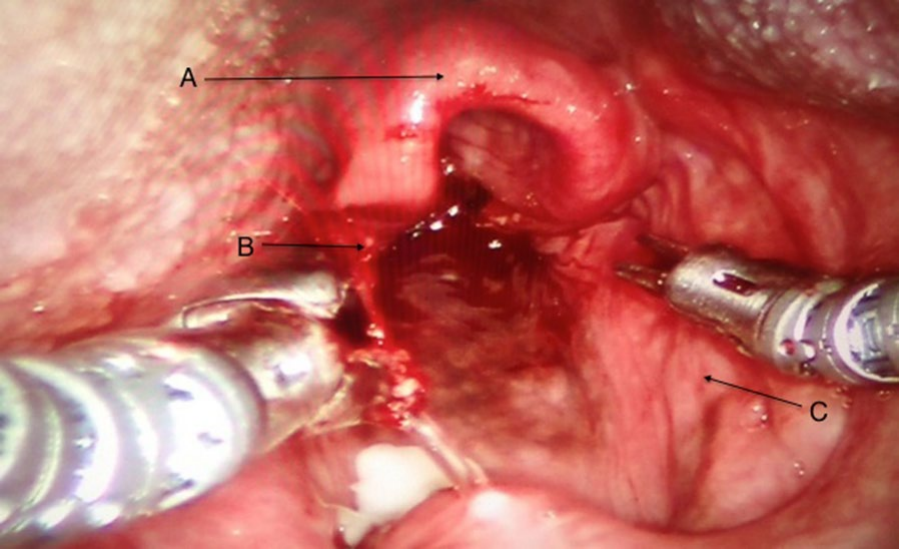
Intraoperative photo demonstrating the robotic arms and exposure attained by the MOR system. *A* epiglottis; *B* aryepiglottic fold; *C* pyriformsinus. 215 × 131 mm (72 × 72)

After general anesthesia was induced and a shoulder roll was inserted, the MOR was inserted into the oral cavity using an appropriate curved blade and was suspended using the MOR suspension block. The retractor gave excellent access to the larynx (Fig. 2). The tongue blade used was a curved type that inserted into the vallecular space. There are multiple blade types available with the MOR system that takes into account the differences in anatomy between patients. When initially inserting the retractor using a headlight, the curve of the tongue blade provided elevation of the epiglottis that exposed the arytenoid mucosa. In some patients, more of the glottis larynx may be seen and these patients are easier candidates for robotic surgery. Following docking of the robot, the entire glottis including the anterior vocal folds could be visualized with the 30° upward directed scope, but more importantly, accessed by the robotic arms (Fig. 3). The improved exposure eliminated the need for a retraction tongue suture. The robot is docked with a Maryland dissector to the left and a needle driver on the right that allows mobilization and use of the OmniGuide CO_2_ laser. Setup using the retractor was relatively simple. The redundant mucosa on the superior aspect of the arytenoid cartilage on the left was excised completely using the laser at 15 W. Unlike access obtained during microlaryngoscopy with a superior to inferior orientation within a narrow scope, tissue was able to be excised using the laser in a side-to-side manner, which is impossible with microlaryngoscopy. The MOR system therefore allows multiple surgical orientation options at the target tissue level. Hemostasis was achieved with the laser and suction cautery. A similar procedure was performed on the left side. The inter-arytenoid area was not treated to avoid contracture. The redundant mucosa could easily be mobilized with the Maryland forceps (Fig. 4). Because he patient had generalized edema, we elected to ablate the false cord on the left side, which was prominent. There were no complications during the procedure. The patient was monitored closely and had undergone multiple debridements of supraglottic tissue, including using a laryngoscope with dysplastic biopsies negative for malignancy. Six months postoperatively, a biopsy was positive for squamous cell carcinoma and he underwent total laryngectomy.

## Discussion

Few in vivo studies have been reported to investigate the efficacy of RMLS. Two studies used the Dingman mouthgag and a 30° scope to attain a working view of the larynx in a mannequin and cadaver, respectively (Hockstein et al. 2005a, b). Lalich et al. invented a retractor and conducted a study in cadavers with adequate access reported, but the retractor has not been trialed in an in vivo human model to date (Lalich et al. 2014). One study required the use of anterior tongue retraction with a 2.0 silk suture, malleable blade to retract the tongue base, and a Lindholm scope to retract the epiglottis. The robotic arms were inserted on either side of the Lindholm scope (Blanco et al. 2011). Resection of T1 glottic cancers have been successful in a small number of studies, but inadequate exposure was a recurrent theme (Park et al. 2009; Byrd and Duvvari 2013; Kayhan et al. 2012; Lallemant et al. 2013). An FK retractor was used in two studies, but the cumbersome nature of the retractor resulted in collision with the robotic arms and limited access to the anterior commissure in both studies. (Dogan et al. 2001; Blanco et al. 2011).

The senior author has used the Dingman, Crow Davis and FK retractor in the past and developed the MOR system as a simpler option with a wider range of applications. These previously described retractors are ideal to address the oropharynx particularly the palatine tonsil region but are limited in their exposure to other regions of the pharynx or larynx. The FK retractor is especially cumbersome in its use and its exposure of the larynx is very limited, especially in visualization of the anterior glottis. The MOR system was developed with two pivot points on the brace and a wide range of blade designs and can replicate the function of a Dingman or Crow Davis retractor. These features allow the user multiple set up options depending on the anatomical area being addressed. Our institution has not required use of the FK or any other retractor since the MOR became available. Unfortunately, direct comparisons between several retractors in a patient have not been performed due to the risk of unnecessary patient injury from insertion to removal of the retractor and significant operative time delay including use of the robot. A cadaver study in the future may have merit in this regard.

**Fig. 5:**
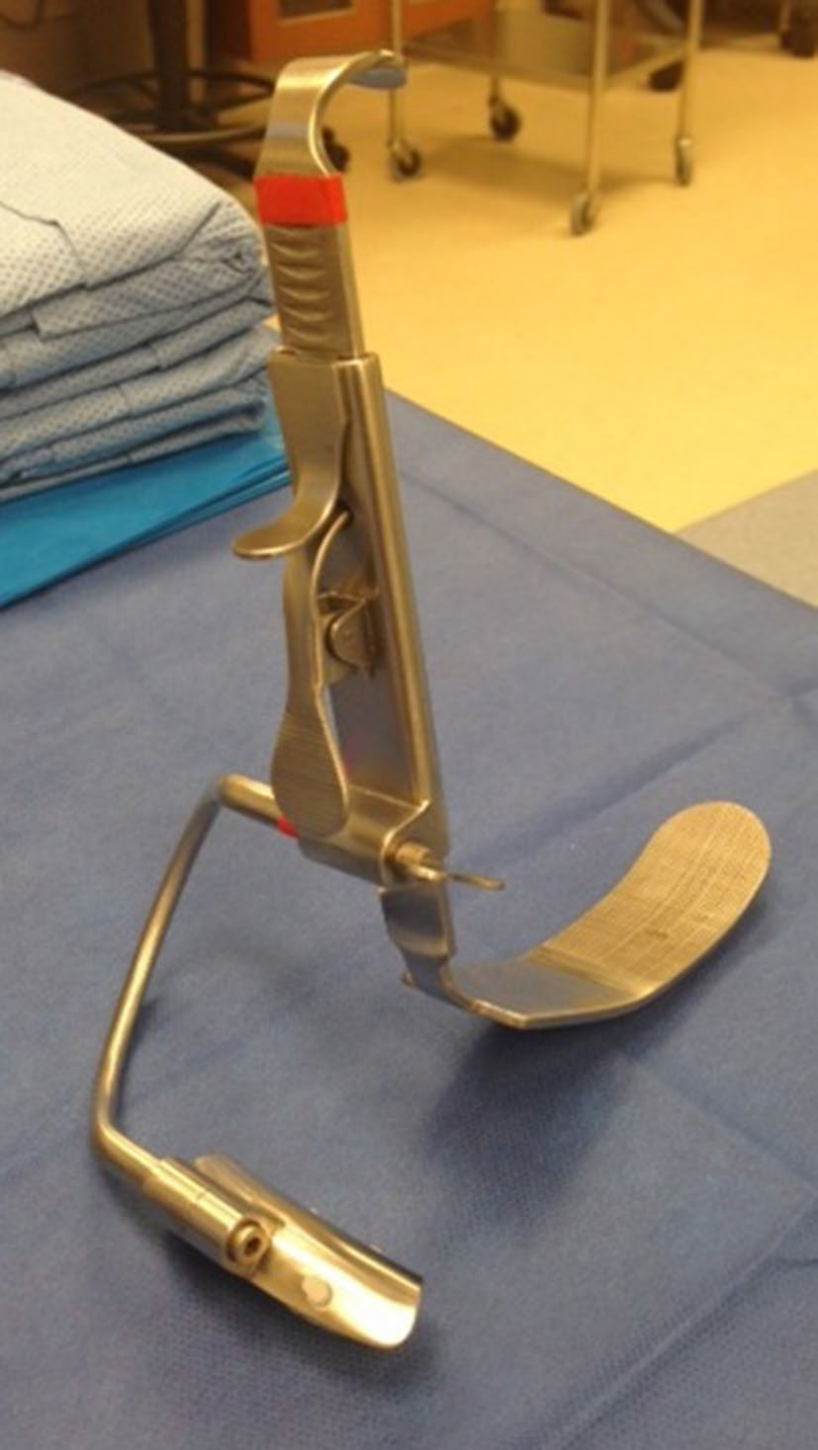
The MOR system displaced with one of the multiple tongue blade options that can be exchanged depending on each patient’s anatomy

**Fig. 6:**
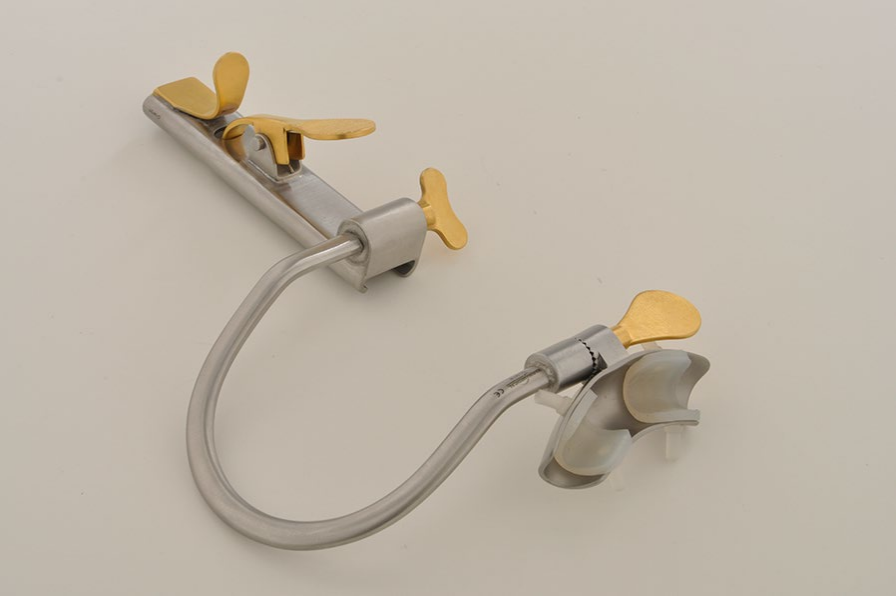
Both the maxillary brace and pivot at the base of the blade have a 360° axis of rotation which improves exposure of the glottis

Our method showed successful robotic-assisted resection of glottic tissue using the MOR system. The MOR system eliminated the need for a rigid circular laryngoscope, which narrows the visual field, increases the distance of the working view to the surgical site and serves as an obstacle around which the robotic arms have to work. The MOR system did not require a stay suture to retract the tongue. Suturing the tongue can cause tongue edema, resulting in limited access and visualization of the surgical site as well as patient discomfort. The retractor has an axis of rotation at the base of the blade that optimizes elevation of the tongue and allows for retraction of the tongue down to the vallecula (Fig. 5). The MOR system includes 24 different blades each of which are customized to overcome commonly encountered anatomical challenges including a large tongue, large base of tongue, or an epiglottis that obscures view of the anterior glottis. The 360° axis of rotation at the pivot of the base of the blade and the inferior portion of the mouth retractor allows excellent exposure of the entire glottis which will likely eliminate the need for a tongue suture in most patients. The curvature of some of the available blades also serves to push the base of tongue anteriorly and allow adequate visualization of the glottis. The maxillary brace also has a 360° axis of rotation that further augments the ability of the retractor to push the tongue and epiglottis forward, allowing visualization of the glottis (Fig. 6). The ability to rotate multiple parts of the retractor around an axis in order to maximize exposure is not possible with other retractors that have been proposed for use in robotic surgery. The robot eliminated tremor as a cause of potential human error and allowed for 360° access to the lesions, which in our case was redundant supraglottic tissue that had prolapsed into the glottic airway.

**Fig. 7:**
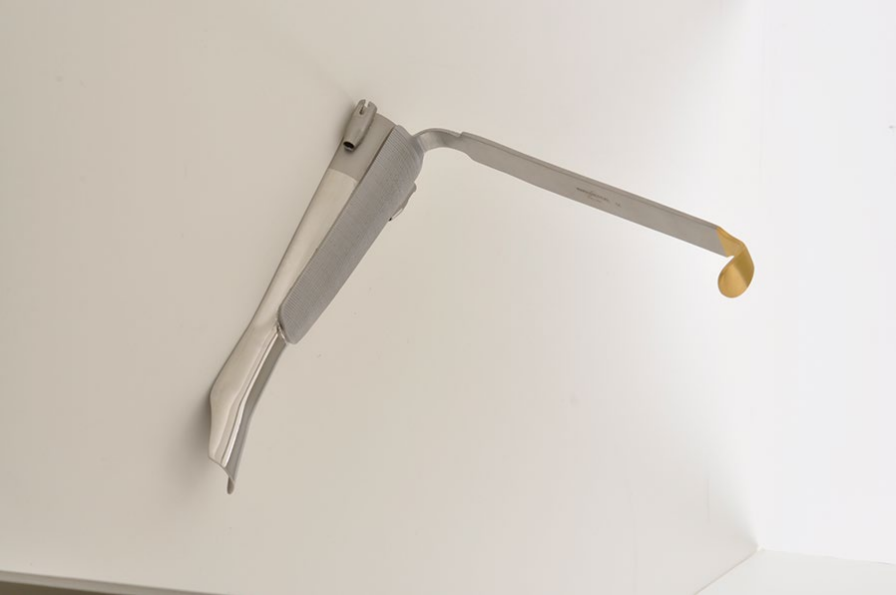
A blade option similar to the superior aspect of a Lindholm blade

Disadvantages of RMLS include limited instrumentation in which success varies depending on each patient’s anatomy, limited tactile feedback requiring the surgeon to rely on visual cues, and limited robotic training programs. Oncologic outcomes using RMLS have not yet been studied or compared to traditional surgery (Byrd and Duvvari 2013). Limitations of this study include that the MOR system has only been evaluated in one case thus far, which limits its generalizability. It has not yet been tested across a wide variety of patient with different anatomy and body habitus. The blade on the retractor does not retract the epiglottis, which is sometimes necessary for exposure depending on the patient’s anatomy. When using the MOR system, attention needs to be given to the size and width of the tongue blade so that the base of the vallecula is reached for optimal glottic exposure. In some cases, the epiglottis may need to be retracted for exposure. We have created multiple blade sizes to address this issue, including a blade similar to the superior aspect of a Lindholm blade that can connect with the MOR brace (Fig. 7). This modification of the Lindholm blade that is commonly used in microlaryngeal surgery gives the robotic surgeon similar access to the larynx. With the recent release of the new da Vinci Xi surgical robot, we believe the MOR system will be even simpler to use with wider application, as the robotic arm instruments are 5 cm longer and more slender.

## Conclusion

RMLS using the MOR system shows promise to allow for easy and effective resection of glottic and supraglottic lesions. We propose that by using this retractor, RMLS may improve upon traditional techniques. Technological advancements such as the new da Vinci Xi robotic system may make the MOR system a better option to perform laryngopharyngeal surgery. Areas of future analysis include a case series of multiple lesions that have been removed via RMLS using the MOR system.
